# 3-dimensional surface geometry dataset of Scots pine and Norway spruce shoots from the Järvselja RAdiation transfer Model Intercomparison (RAMI) pine stand

**DOI:** 10.1016/j.dib.2024.110543

**Published:** 2024-05-18

**Authors:** Jan Pisek, Oleksandr Borysenko

**Affiliations:** a University of Tartu, Tartu Observatory, Observatooriumi 1, 61602 Tõravere, Tartumaa, Estonia; b National Aerospace University, Kharkiv Aviation Institute, Kharkiv, Ukraine

**Keywords:** Shoot structure, GOM photogrammetry, Radiative transfer, Model benchmarking, 3D virtual plant canopy, RAMI

## Abstract

Conifer shoots exhibit intricate geometries at an exceptionally detailed spatial scale. Describing the complete structure of a conifer shoot, which contributes to a radiation scattering pattern, has been difficult, and the previous respective components of radiative transfer models for conifer stands were rather coarse. This paper presents a dataset aimed at models and applications requiring detailed 3D representations of needle shoots. The data collection was conducted in the Järvselja RAdiation transfer Model Intercomparison (RAMI) pine stand in Estonia. The dataset includes 3-dimensional surface information on 10 shoots of two conifer species present in the stand (5 shoots per species) - Scots pine (*Pinus sylvestris* L.) and Norway spruce (*Picea abies* L. *Karst.*). The samples were collected on 26th July 2022, and subsequently blue light 3D photogrammetry scanning technique was used to obtain their high-resolution 3D point cloud representations. For each of these samples, the dataset comprises of a photo of the sampled shoot and its obtained 3-dimensional surface reconstruction. Scanned shoots may replace previous, artificially generated models and contribute to the more realistic representation of 3D forest representations and, consequently, more accurate estimates of related parameters and processes by radiative transfer models.

Specifications Table

**Table:** 

Subject	Plant Physiology and Ecological Modelling
Specific subject area	Anatomy; ecophysiology; conifer shoot; radiative transfer modelling; remote sensing
Type of data	3-dimensional surface geometries of the sampled conifer shoots from GOM scanner data (.stl for 3-dimensional surface geometry exchange format) Photos of the in sampled conifer shoots (.jpg for JPEG format)
Data collection	The Scots pine (*Pinus sylvestris* L.) and Norway spruce (*Picea abies (*L.*) Karst.*) shoots were scanned with an industrial non-contact 3D scanner GOM Scan 1 (MV 200 version) (Carl Zeiss GOM Metrology GmbH, Germany). The shoots came from branches that were collected using a telescopic tree pruner from the understory Norway spruce trees and Scots pine trees in overstory in the RAMI Järvselja pine stand on 26th July 2022. Five shoots per species with different shoot lengths, needle densities were cut from the branches and scanned in the laboratory.
Data source location	Localization: The RAdiative transfer Model Intercomparison (RAMI) Järvselja Pine Stand Country: Estonia Latitude and longitude: 58° 18′ 47.13″ N, 27° 17′ 48.23″ E
Data accessibility	Repository name: Mendeley Data identification number: 10.17632/rs3f6trdvw.1 Direct URL to data: https://data.mendeley.com/datasets/rs3f6trdvw/1
Related research article	Pisek, J., Borysenko, O., Janoutová, R., Homolová, L., 2023. Estimation of coniferous shoot structure by high precision blue light 3D photogrammetry scanning. Remote Sensing of Environment 291, 113568. https://doi.org/10.1016/j.rse.2023.113568

## 1 Value of the Data

• Conifer shoots exhibit intricate geometries at an exceptionally detailed spatial scale. Describing the complete structure of a conifer shoot, which contributes to a radiation scattering pattern, has been difficult, and the previous respective components of radiative transfer models for conifer stands were rather coarse.
• The dataset contains 10 high-resolution reconstructed surface models of conifer shoots (Scots pine, Norway spruce) collected in the Järvselja RAdiation transfer Model Intercomparison (RAMI) pine stand in Estonia.
• Provided shoot models reconstructed from actual 3D point clouds may replace artificially generated models that were used in previous phases of the RAMI exercise.
• Detailed 3D representations of included needle shoots may bring many opportunities for further improvements of measurement methods in the upscaling of optical properties for coniferous canopies.
• Provided 3D models of conifer shoots can be used to analyze the morphology of shoots to understand factors such as needle arrangement, shoot structure, or overall shape, which can provide insights into tree health, productivity, and response to environmental conditions.
• Besides forestry and ecology research, the included conifer shoot representations can be integrated into a generation of virtual trees, forests, and environmental simulations, their realistic renderings, and visualizations for various purposes, including learning tools to increase their realism and authenticity.

## 2 Background

The reported dataset was originally collected to explore if 3D photogrammetry scanning can reduce the labor intensity of the previous approaches to estimate foliage clumping within a shoot [1], where foliage clumping within a shoot describes the spatial arrangement of individual needles within a shoot [2]. This data article adds value to [1] by making the created dataset publicly available and usable for other purposes beyond foliage clumping estimation and potentially helping to improve coniferous forest 3D radiative transfer modelling in general.

## 3 Data Description

**Fig. 1:**
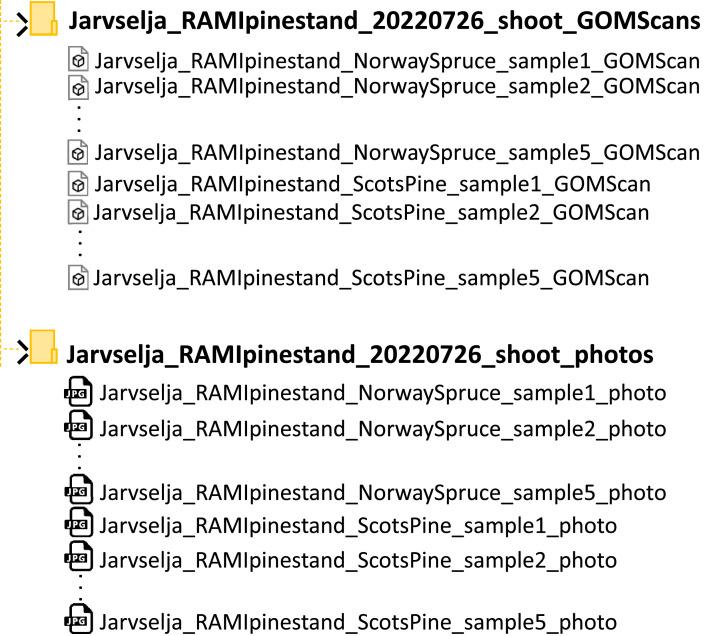
Dataset files and folders structure.

The dataset can be downloaded from https://data.mendeley.com/datasets/rs3f6trdvw/1. It consists of two compressed folders. Once unpacked, the data appear as shown in Fig. 1. The first folder contains five 3-dimensional surface geometry shoot models per given species (.STL format). The second folder contains a photo (.JPG format) of each scanned sample shoot. The dataset's size amounts to 765 megabytes while uncompressed.

## 4 Experimental Design, Materials and Methods

The Järvselja RAMI pine stand [3] (latitude 58° 18′ 47.13′′ N; longitude 27° 17′ 48.23′′ E), primarily comprises Scots pine (*Pinus sylvestris* L.) trees in the overstory, (average tree height 17.4 m), with occasional small (*H* < 2 m) isolated Norway spruce (*Picea abies (*L.*) Karst*) trees in the understory [4]. On July 26th, 2022, branches with shoots from both conifer species were collected using a telescopic tree pruner. In the laboratory, five shoots per species, featuring varying shoot lengths and needle densities, were cut from the collected branches. An industrial non-contact 3D scanner, GOM Scan 1 (MV 200 version; Carl Zeiss GOM Metrology GmbH, Germany) was used to scan the shoots. The setup also included a GOM ROT 350 automatic rotation table connected to a laptop, all managed by GOM Software – GOM Inspect v2.0.1 (Rev.151410).

**Fig. 2:**
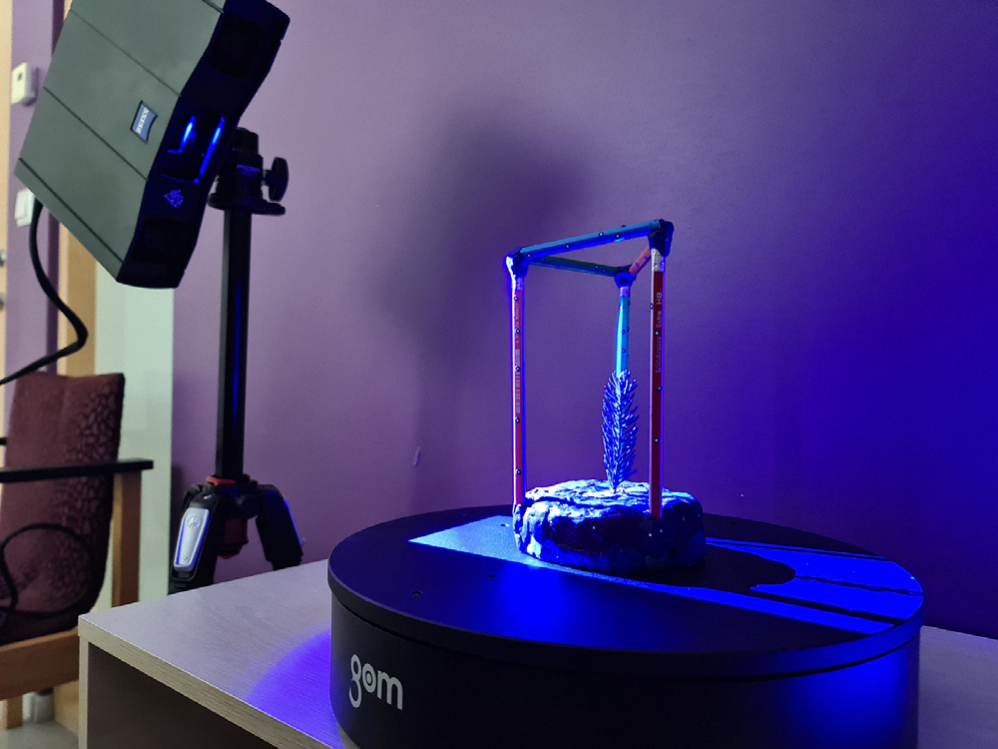
Measuring set up for the blue light 3D scanning with GOM Scan 1 (MV 200 version). The sample is placed inside the custom frame (mode of wooden sticks here) in the center of the rotating table. The frame is covered with reference point stickers to achieve accurate co-registration of point clouds along the full sample height profile. The scanner captures data from various sides of the sample during the scanning process. The whole scanning process and the individual components (scanner, rotating table) are controlled by the computer.

**Fig. 3:**
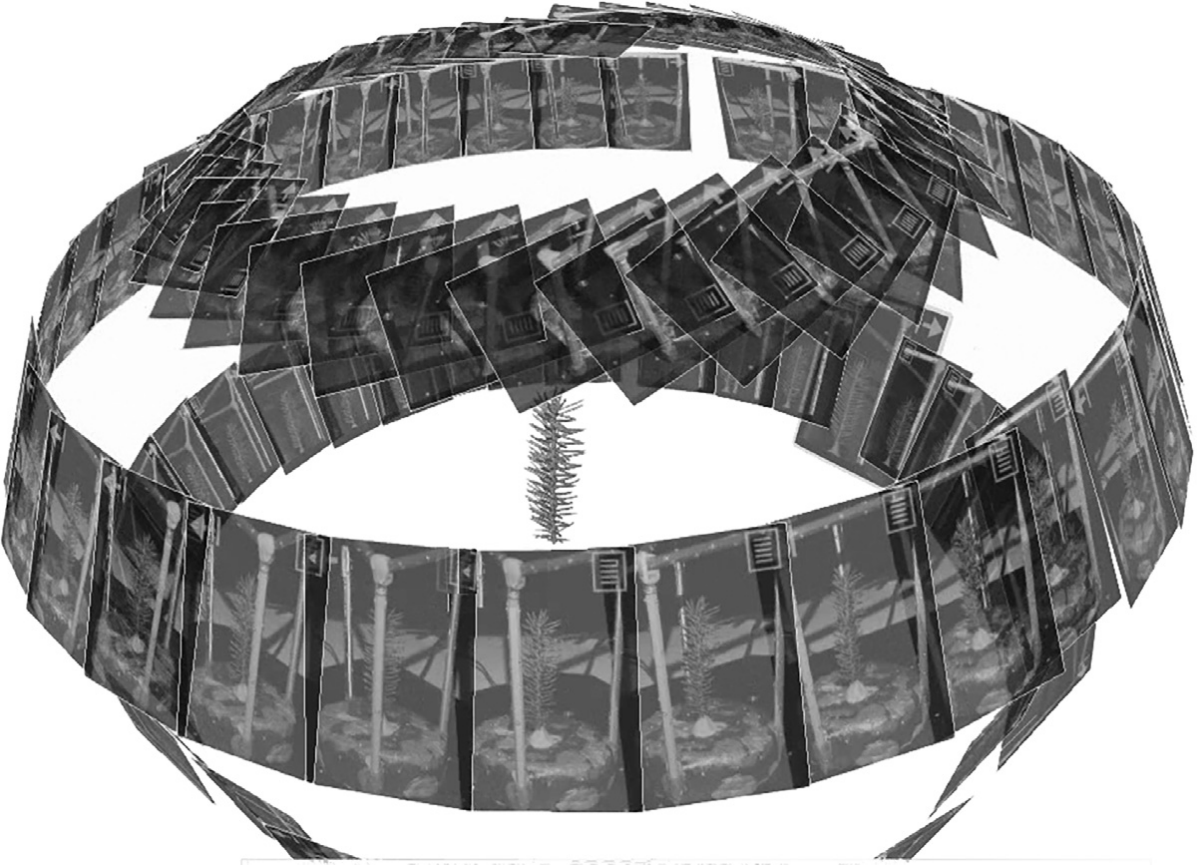
Example of the collected photos and scanner positions to obtain the 3D point cloud representations of the sampled shoots.

The shoots were affixed to the rotation table with plasticine clay at their base to maintain an upright position. The whole setup is shown in Fig. 2. Each shoot sample underwent scanning at three different view zenith angles: 30°, 60°, and 100°. The scanner was positioned horizontally for the 30° view and vertically for the 60° and 100° views to ensure comprehensive scanning coverage and optimal co-registration. Fig. 3 presents the example of all the collected photos and scanner positions to obtain the 3D point cloud representation of the given shoot. The number of scans differed depending on the complexity of the shoots, but generally increased with shoot height and needle distribution. A custom frame facilitated scanning accuracy and co-registration by allowing the placement of reference point stickers around the shoots. The stability of transformations was ensured with a transformation error of less than 0.009 mm. To prevent undesired movement during rotation, a monofilament hook line with a 0.09 mm diameter stabilized the shoots.

**Fig. 4:**
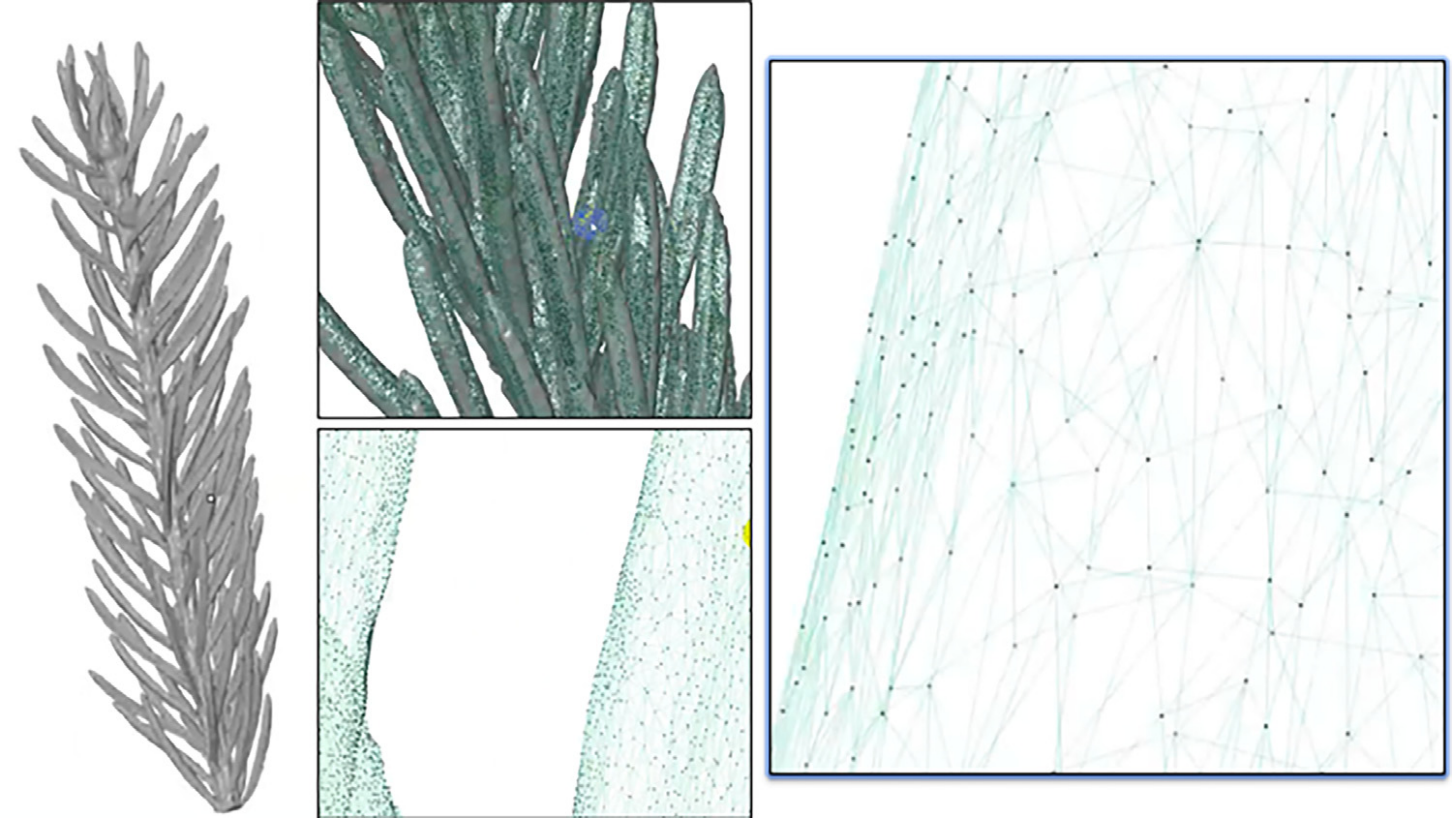
Final reconstruction of a needle shoot, close-up look of the collected point cloud, and reconstructed surface area for one of the Norway spruce shoot samples.

**Fig. 5:**
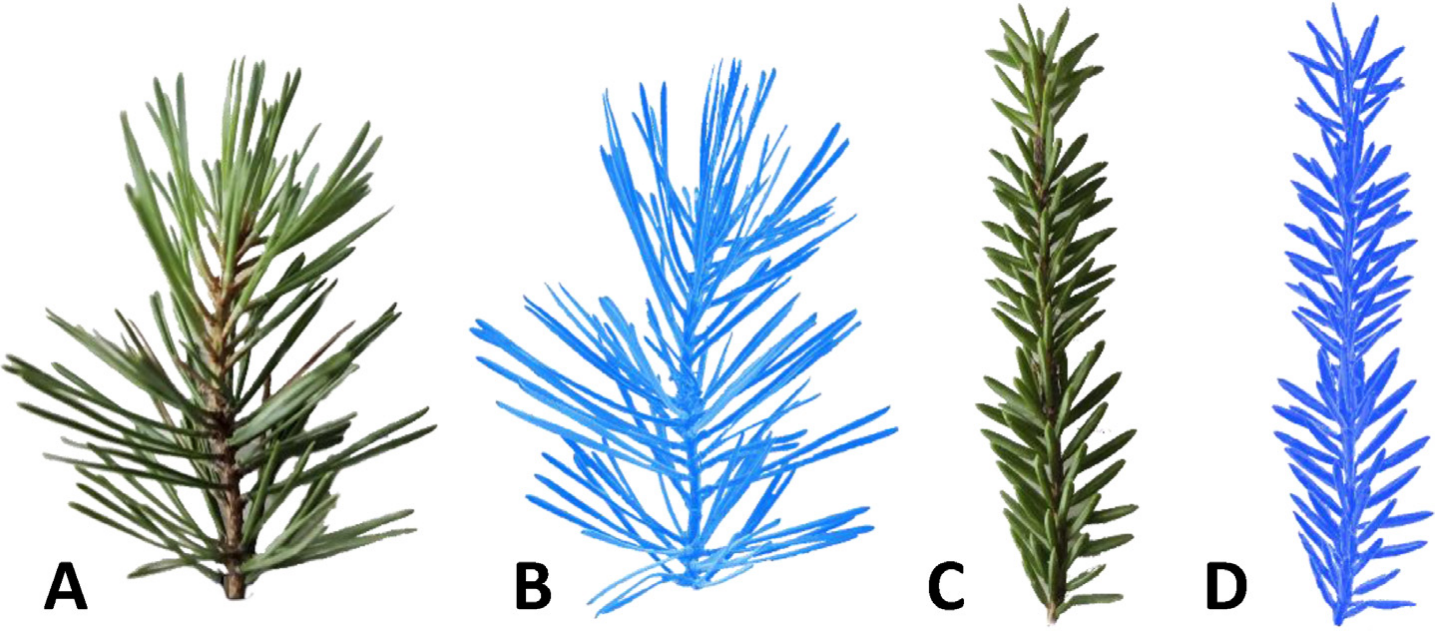
Comparison between actual shoots and corresponding reconstructed 3D surfaces for sampled Scots pine (sample 2) (A,B) and Norway spruce (sample 3) (C,D) shoots.

Different exposure times were determined for optimal light capture, with exposure times set at 16.81 ms and 27.67 ms to scan the target, and 8.40 ms for reference points. The More Points Option in GOM Inspect Software was chosen for scan point computation due to its enhanced post-processing solutions compared to the Best Fit Option. Background cut-out was disabled during scanning. Between 90 and 180 scans were conducted per shoot, based on size and complexity, with the final number determined by visual inspection to ensure comprehensive coverage. Following scanning and background removal, any remaining gaps in the models were manually closed using available interactive tools in GOM Inspect software. The resulting models, containing between 0.5 M to 1.3 M points each (Fig. 4), were exported in .stl format and provided in the dataset. Fig. 5 offers examples of the actual and reconstructed shoots included in the dataset for each species.

## Limitations

There are 971 Scots pine trees within the 1-ha RAMI plot. As such, the five surface models of Scots pine included in the dataset may not be rigorously representative of the full distribution of architectural shoot types in the plot. Also, due to the limited length and reach of the telescopic tree pruner, the collected branches came mostly from the lower crown sections of Scots pine overstory in the stand.

## Ethics Statement

The authors declare that this work did not involve human subjects nor animal experiment nor data collected from social media platforms. They have read and followed the ethical requirements for publication in Data in Brief journal.

## CRediT authorship contribution statement

**Jan Pisek:** Conceptualization, Methodology, Data curation, Writing – original draft, Funding acquisition. **Oleksandr Borysenko:** Visualization, Investigation.

## Data Availability

A dataset of detailed 3D representations of Scots pine, Norway spruce needle shoots from Järvselja RAdiation transfer Model Intercomparison (RAMI) pine stand in Estonia (Original data) (Mendeley Data)
